# Differential Effects of Pandemic-Related Stressors on Mental Health by Age and Sex

**DOI:** 10.3390/healthcare13030224

**Published:** 2025-01-23

**Authors:** Joshua B. Borja, Scott B. Patten

**Affiliations:** 1 Department of Community Health Sciences, Cumming School of Medicine, University of Calgary, 3280 Hospital Dr. NW, Calgary, AB T2N 4Z6, Canada; 2 Mathison Centre for Mental Health Research & Education, Hotchkiss Brain Institute, Cumming School of Medicine, University of Calgary, 3280 Hospital Dr. NW, Calgary, AB T2N 4Z6, Canada

**Keywords:** youth, mental health, major depressive episode, generalized anxiety disorder, distress, sex, COVID-19, population health

## Abstract

Objective: There have been consistent concerns about a greater impact of COVID-19 on the mental health of younger people and females. We aimed to explore the potential synergistic effect of various pandemic-related stressors with age and sex on the mental health of the general Canadian household population during the COVID-19 pandemic. Methods: Using cross-sectional data from the Statistics Canada 2022 Mental Health and Access to Care Survey (MHACS), frequencies for major depressive episode (MDE), generalized anxiety disorder (GAD), general psychological distress, and various pandemic-related and demographic factors were estimated. Odds ratios were estimated using binary logistic regression models. These estimates used a replicate bootstrapping procedure recommended by Statistics Canada. Finally, Relative Excess Risk due to Interaction (RERI) models were used for each outcome to evaluate the interactions of each pandemic-related stressor with age and sex on an additive scale. Results: Past-12-month MDE and GAD, psychological distress, and the various COVID-19 stressors were more prevalent in young people and females. Overall, the stressors were confirmed to be associated with these outcomes. There were greater-than-additive interactions between age and difficulty accessing healthcare, loneliness, physical health problems, household relationship challenges, and work stress; and between sex and severe illness of a loved one, loneliness, work stress, LGBTQ2+ status, marital status, difficulty accessing healthcare, physical health problems, job/income loss, and financial difficulties. Generally, evidence of synergy was found between age and pandemic-related stressors and sex and pandemic-related stressors. Conclusions: Greater-than-additive interactions of pandemic-related stressors with age and sex may indicate synergistic vulnerabilities within females and young people. Future pandemics, via corresponding stressors, may be associated with increased mental health vulnerability in females, youth, and especially young females.

## 1. Introduction

In March 2020, the World Health Organization declared the novel Coronavirus disease 2019 (COVID-19) a global pandemic [1]. Many jurisdictions worldwide imposed restrictions intended to reduce the spread of the virus, such as travel restrictions, social distancing, quarantining, stay-at-home orders, and school and business closures. These may have contributed to the increased global prevalence of mental health problems [2]. The public health response may have contributed to emotional reactions, including distress and psychiatric conditions [3]. Authors have consistently stated concerns about a greater impact on the mental health of younger people or females [4,5,6,7,8,9,10,11,12,13,14]. However, almost all studies in the existing literature have relied on brief symptom scales rather than diagnostic measures, and non-representative convenience samples. Due to the lack of diagnostic assessment, and other methodological issues such as a focus on risks rather than excess risk, these studies have not been able to make any firm conclusions about increased vulnerability among these groups. By examining patterns of prevalence of major depressive episodes (MDEs), generalized anxiety disorder (GAD), and general psychological distress, we aim to confirm the hypothesis that younger people and females may be more vulnerable to MDE, GAD, and general psychological distress when exposed to pandemic-related stressors.

Internationally, an increase in depressive and anxiety symptoms among children and adolescents has been reported during the pandemic. A systematic review of this literature found that the frequency of exceeding cut-points on symptom scales was 25.2% for depression and 20.5% for anxiety in children and adolescents aged less than 18 years [15]. These frequencies, however, do not reflect the prevalence of mood and anxiety disorders and may represent self-limited stress-related symptoms, such as adjustment disorders. After comparing with pre-pandemic studies, these authors concluded that the risk of elevated symptoms had doubled. Conversely, a study from the Netherlands that used a diagnostic measure found no difference in the prevalence of depression or anxiety disorders after the onset of the pandemic [16].

Younger individuals reported significantly higher levels of anxiety and depression compared to older age groups during COVID-19 [10,12,13,17,18], and females have consistently reported higher levels of anxiety, depression, and stress than males [7,19,20,21,22,23]. However, this does not necessarily reflect a change in disorder prevalence, since symptoms of depression and anxiety tend to be generally higher in young adults and women. For younger age groups, authors have speculated that psychosocial stressors such as social isolation and loneliness, economic uncertainty, concerns about physical health, and the disruption of daily routines had exacerbated the prevalence of adverse mental health outcomes [10,24]. Notably, older adolescents exhibited higher rates of depression compared to younger children, which Racine et al. attribute to social isolation, puberty and hormonal changes, and a lack of socialization with peers [15]. Most young adults (60%) have also reported adverse pandemic-related effects on their physical, emotional, and social well-being [13]. Similarly, most studies have speculated about potential causes of sex differences, such as Hawes et al. [19], who describe the increased caregiving responsibilities, greater impact of social isolation, and heightened sensitivity to health-related anxieties as potential explanations for these sex differences.

The risk factors associated with poor mental health outcomes during the COVID-19 pandemic are diverse, with various social, psychological, physical, and financial stressors linked to the increased prevalence of mood and anxiety symptoms. Economic challenges were among the largest sources of stress and were found to be significantly associated with higher levels of depression, anxiety, and stress [25]. Such challenges include lower socioeconomic status and having fewer assets [26,27], income loss and greater financial strain [28], and unsteady income or unemployment [9]. Disruptions in access to childcare and healthcare services have also contributed to poor mental health outcomes [29,30]. Additionally, inadequate access to medications also emerged from the public health measures [31]. Social isolation and loneliness have been major stressors during the pandemic, with feelings of loneliness being strongly correlated with depression and anxiety symptoms [32,33]. Feelings of loneliness were especially high among young adults and females [34,35,36]. Other factors associated with increased rates of depression and anxiety symptoms include problems with relationships [8,37], the death of a loved one [38], and chronic pain [39].

Certain demographic factors have also been shown to predict higher levels of adverse mental health outcomes during the pandemic. LGBTQ2+ individuals reported higher levels of depression, anxiety, and alcohol use compared to cisgender straight individuals [40]. Multiple studies have shown low education status as a predictor of poor mental health [8,26,31,32]. Work stress during the pandemic is one risk factor that has been scarcely studied, though it has been shown to be associated with major depressive episodes [41,42]. The aforementioned factors, however, are associated with depression and anxiety even when without a pandemic.

There remains a need for further research to appropriately quantify how these various stressors may interact with age and sex to exacerbate mental health issues among young people and females during a pandemic like COVID-19. This idea needs to be evaluated using statistical interactions, specifically on an additive scale, as greater-than-additive interactions are more directly linked to causation [43]. A greater-than-additive effect of a joint exposure implies, subject to the inherent limitations of cross-sectional data, that two characteristics have a synergistic effect in combination because it implies the existence of causal mechanisms that require both exposures [44].

Our objective was to provide epidemiologic confirmation of the greater vulnerability of young people and females to developing mental health problems when exposed to pandemic-related stressors. Quantifying vulnerability in relation to age and sex and highlighting the differential effects of pandemic-related stressors have important implications for public health decisions that will be a part of risk–benefit calculations in future public health emergencies. Thus, this study aimed to explore (1) the prevalence of MDE, GAD, and general psychological distress in the Canadian general household population during the pandemic period, and (2) the potential synergy between various pandemic-related stressors and demographic factors with (a) age, focusing on the 15–24 age group, and (b) sex, focusing on females.

## 2. Materials and Methods

### 2.1. Data Source

The data source for this study was the Mental Health and Access to Care Survey (MHACS), conducted by Statistics Canada from March to July 2022 [45]. The MHACS user guide, questionnaire, and data dictionary can be publicly accessed at https://www150.statcan.gc.ca/n1/en/catalogue/82M0021X (accessed on 18 December 2024). MHACS was a cross-sectional study that included a fully structured diagnostic interview, a Canadian adaptation of the World Mental Health Composite International Diagnostic Interview (CIDI), administered via Computer Assisted Telephone Interviewing (CATI). The same interview was used in prior national mental health surveys in Canada in 2002 and 2012 [46,47,48]. The MHACS included measures of various pandemic-related stressors and demographic variables.

The population under study includes people aged 15 years and over living in private dwellings in the ten Canadian provinces, framed from the set of respondents identified in the 2021 Canadian census [45]. The initial sample size of 40,000 households was stratified into age (15–24, 25–44, 45–64, and 65+), sex (female and male), and population groups (South Asian, Black, Chinese, Filipino, and Other) [45]. This multi-phase sampling allowed Statistics Canada to collect enough data to produce precise estimates at the national level for each stratum [45], though deviations from simple random sampling, including oversampling and unequal selection probabilities, arise from the survey design. A simple random sample of households was selected independently within each stratum, with each respondent assigned a survey weight corresponding to a certain number of persons in the Canadian population [45]. After two stages of selecting a household and an individual from each selected household, the final sample was 25% of the initial 40,000, a lower response rate than previous Canadian national mental health surveys. An effort was made to minimize bias due to non-response through the weighting strategies applied (which included adjustments for non-response) [45]. Sampling weights were adjusted to offset non-response by increasing the weights proportionally to the lack of response in various demographic groups. Through these strategies and weight adjustments, Statistics Canada has determined that the Canadian population was well-represented when compared to the 2021 census. It is important to emphasize that no data was collected within Indigenous reserves, other Aboriginal settlements, the Canadian Forces, or collective dwellings, such that the results should not be generalized to these groups. Certain remote areas were also excluded from the sampling frame [45]. In their totality, these exclusions amounted to approximately 3% of the national population [45].

### 2.2. Measures

#### 2.2.1. Mental Health Outcomes

The outcomes of interest include a positive result for past-12-month MDEs and past-12-month GAD using the CIDI, and general psychological distress using the Kessler Psychological Distress Scale (K10). The CIDI is a critical component of the MHACS design, given its ability to generate mental disorder diagnoses consistent with the Diagnostic and Statistical Manual of Mental Disorders, Fourth Edition (DSM-IV) [49]. The WMH-CIDI is a structured interview and is not scored with a cut-off but rather with an algorithm reflecting the DSM-IV diagnostic criteria. The K10 provides a range of scores of 0 to 40, and the cutoff for a positive distress result used in the current analysis was a score of ≥20, given that it indicates a mental disorder is likely present [50]. The K10 is significantly associated with CIDI diagnoses for mental disorders [50], and provides a broader indication of mental disorder prevalence beyond the two specific diagnostic categories examined. Cronbach’s alpha for the K10 distress scale for psychological distress was 0.89 [51].

#### 2.2.2. Age and Sex

This study focused on young people and females. The definition of young people in this study is individuals aged 15–24, the youngest age group stratified by Statistics Canada. The 15–24 age group was chosen for our study because emerging adulthood is a period of peak vulnerability for the onset of mental health disorders [52]. Sex was a binary gender variable derived from Census responses, through which Statistics Canada classified each respondent as either a man or woman [45].

#### 2.2.3. Pandemic-Related Stressors

We used a series of yes/no MHACS items assessing pandemic-related stressors, with each stressor emerging during COVID-19. In the MHACS questionnaire [45], respondents were asked if they were affected by the following experiences of COVID-19 impacts:

1. Loss of job or income;
2. Difficulty meeting financial obligations or essential needs (e.g., rent or mortgage payments, utilities, and groceries);
3. Difficulty accessing required childcare services;
4. Difficulty accessing required medications;
5. Difficulty accessing required health care services;
6. Diagnosed with COVID-19;
7. Hospitalized due to COVID-19;
8. Severe illness of a family member, friend, or someone you care about;
9. Death of a family member, friend, or someone you care about;
10. Feelings of loneliness or isolation;
11. Emotional distress (e.g., grief, anger, worry, etc.);
12. Physical health problems (e.g., weight gain or loss, high blood pressure, headaches, sleep problems, etc.);
13. Challenges in personal relationships with members of your household (e.g., children, spouse, parent, grandparents, etc.);
14. Other;
15. None of the above.

Statistics Canada justifies these items as necessary to collect data on factors associated with mental health [45], representing the pandemic’s major impacts. Item 7 was omitted as there were insufficient data for the 15–24 group, such that the analysis could not accommodate the inclusion of hospitalization among young people. Emotional distress is considered a component of the mental health outcomes in our analysis, not an exposure or risk factor; thus, item 11 was omitted to prevent reporting of a tautological association. Item 14 was deemed too vague with an unspecified and unclear meaning, and item 15 was considered uninterpretable. We also created an additional variable to measure the presence of ‘any’ COVID-19 impact by combining the items chosen for inclusion.

#### 2.2.4. Other Factors

Demographic characteristics were also included in the analysis. A variable for low family income was created by taking the lowest decile in a derived variable linked from tax records from the 2021 census that accounts for family size and place of residence. Marital status was stratified by married or common law versus never married, separated, divorced, or widowed. Other demographic factors included membership in the LGBTQ2+ community, being an immigrant, and having less than a postsecondary education. Variables for chronic pain and physical inactivity were also created.

### 2.3. Statistical Analysis

Stata 18 (version 18.5, StataCorp LLC, College Station, TX, USA) was the software used for the data analysis [53], which took place at the Prairie Regional Research Data Centre at the University of Calgary from May to June 2024. The prevalence of each binary outcome and stressor was estimated across five population groups: (1) total population, (2) 15–24 age group, (3) 25+ age group, (4) females, and (5) males. Detailed descriptive statistics of the MHACS sample have been reported by Statistics Canada (see Stephenson) [54]. We used survey proportion estimations with 1000 bootstrap replications, rescaled using a Fay adjustment of 0.67, as recommended by Statistics Canada, to obtain frequency estimates for each group, along with 95% confidence intervals (CIs) [45]. The set of replicate bootstrap weights provided by Statistics Canada accounts for the design effects described above (unequal selection probabilities and clustering arising from the multiple sampling stages). The next step of the analysis was calculating odds ratio estimates using similarly bootstrapped binary logistic regression models. Each stressor was added to the three separate outcomes to confirm that they were indeed risk factors for MDEs, GAD, and distress. Subsequently, Relative Excess Risk due to Interaction (RERI) models were used to estimate greater-than-additive interactions in logistic regressions. The estimation of RERIs in Stata used the “master” sampling weights, helping to ensure the validity of the point estimates, but could not accommodate replicate bootstrap weights. Therefore, the standard error associated with each RERI may be underestimated as clustering is not addressed. The interaction of each stressor and age and the interaction of each stressor and sex on an additive scale was computed. The 95% CIs were used to characterize the potential range of population values, acknowledging that due to clustering, the 95% CIs may be narrower than their true values. A RERI with lower confidence limits that exceed 0 indicates a greater-than-additive interaction at the 95% level of confidence, and a RERI whose CIs are less than 0 indicates that the data are consistent, at the 95% level of confidence, with a null hypothesis of no additive interaction. Because the RERI has a defined null value of 0, overlap of the 95% CIs with this value provides an assessment of significance at the 95% level of confidence. *p*-values are not reported since the 95% CIs provide the same information and more, such as the level of precision attained.

## 3. Results

The descriptive features of the general Canadian household population, as represented by the MHACS sample of n = 9861, are presented in Table 1. The response rate was 25% of the original n = 39,485 households. Frequency estimates for each mental health outcome, demographic characteristics, COVID-19 impacts, and other stressors by population group are reported in Table 2. Nationally, the prevalence of MDE, GAD, and general psychological distress was 7.6%, 5.2%, and 6.3%, respectively. A total of 75.7% of Canadians experienced at least one COVID-19 impact.

The younger age group reported higher rates of all three outcomes than the older age group, such that MDE, GAD, and distress were generally twice as prevalent in young people. Being in the younger age group was associated with MDE (OR 2.2; 95% CI 1.8–2.7), GAD (OR 1.9; 95% CI 1.5–2.3), and distress (OR 2.0; 95% CI 1.6–2.5). Young people reported higher levels of job/income loss, COVID-19 diagnoses, loneliness, and relationship challenges with household members than those aged 25+. Overall, pandemic-related stressors were more prevalent among young people, with 81.0% reporting an experience of at least one COVID-19 impact, compared to 74.8% in the 25+ group. The 25+ group experienced higher rates of all three difficulties related to access to care (childcare, medications, and healthcare) and the death of a loved one. There were similar frequencies of financial difficulties, severe illness of a loved one, and physical health problems between the two age groups. The 25+ group experienced higher rates of work stress, chronic pain, and physical inactivity.

Table 3 shows the odds ratios (ORs) indicating the impact of the various demographic, pandemic-related and other stressors on the prevalence of MDE, GAD, and general psychological distress. For the demographic characteristics, associations with MDE, GAD, and distress were seen for a young age, being female, low family income, and being an LGBTQ2+ person. Low education was associated with MDE and distress but not GAD. Marital status and immigrant status did not have an association with any outcome. Across the demographic stressors, LGBTQ2+ status had the strongest association with MDE, GAD, and distress. Work stress and physical inactivity were associated with the three outcomes, and chronic pain was associated with MDE and distress only.

The various COVID-19 impacts were confirmed to be associated with and thus risk factors for the three outcomes, albeit with two exceptions. A COVID-19 diagnosis was associated with GAD but not MDE or distress. The death of a loved one was associated with MDE and distress but not GAD. Economic challenges and difficulties related to access had an association with all three outcomes. Experiences of a loved one’s severe illness also showed an association. The strongest association was found between loneliness and GAD. Moreover, loneliness was found to have the strongest associations across MDE, GAD, and distress. Physical health problems and relationship challenges with household members also had strong associations. An experience of any pandemic-related stressor was associated with all three adverse mental health outcomes.

**Figure 1:**
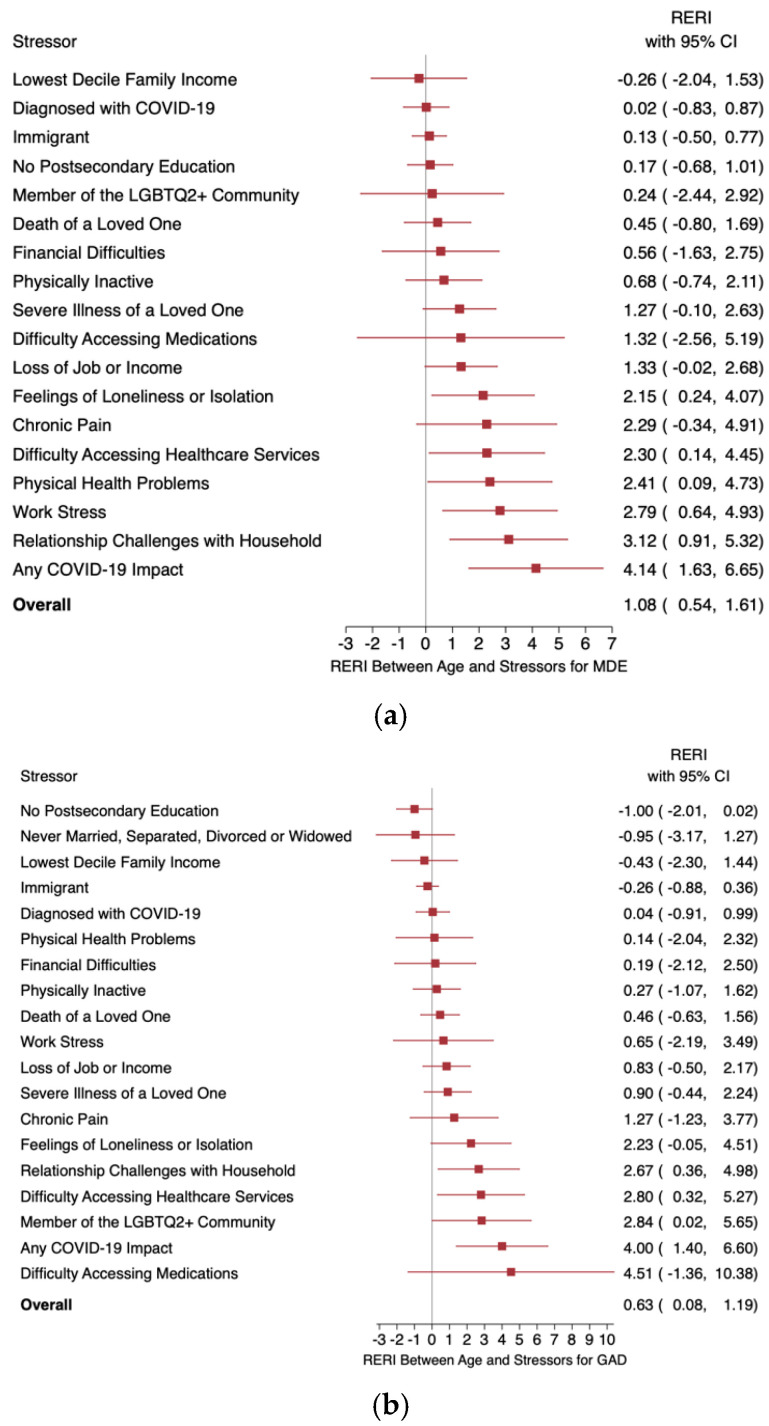

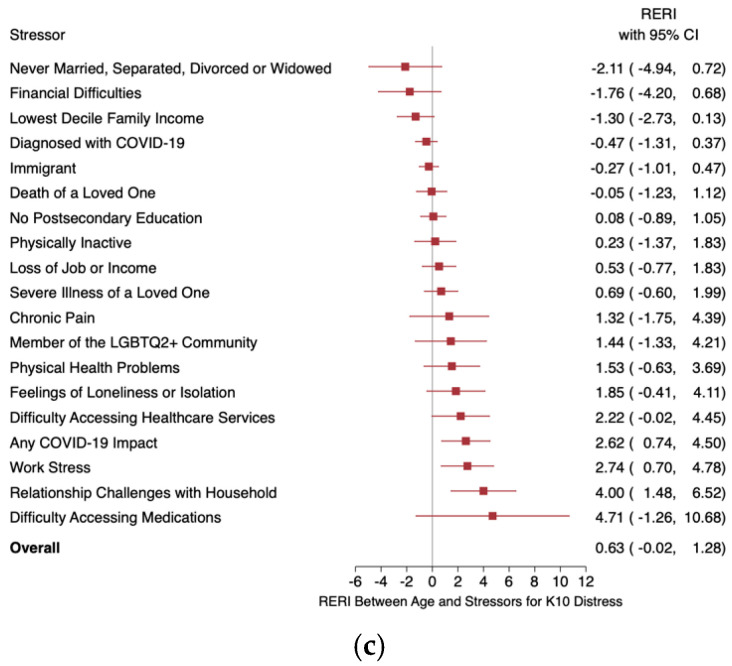
Forest plots summarizing the RERIs between age and various pandemic-related stressors for (**a**) MDE, (**b**) GAD, and (**c**) K10 distress. Red squares represent the point estimates for the RERIs between age and pandemic-related stressors; horizontal bars represent 95% CIs.

The RERIs between age and each stressor for MDE, GAD, and distress are summarized in forest plots in Figure 1 and presented in more detail in Table 4. For MDE, there was a significant greater-than-additive interaction between age and the following stressors: difficulty accessing healthcare, loneliness, physical health problems, work stress, and relationship challenges with household members. For GAD, significant greater-than-additive interactions with age were found for relationship challenges with household members, difficulty accessing healthcare, and LGBTQ2+ status. For K10 distress, significant greater-than-additive interactions with age were shown for work stress and relationship challenges with household members. Greater-than-additive interactions between difficulty accessing healthcare services, relationship challenges with household members, work stress, and LGBTQ2+ status with age were consistent across at least two outcomes. The RERI model for household relationship challenges yielded a greater-than-additive interaction with age across all three outcomes.

**Figure 2:**
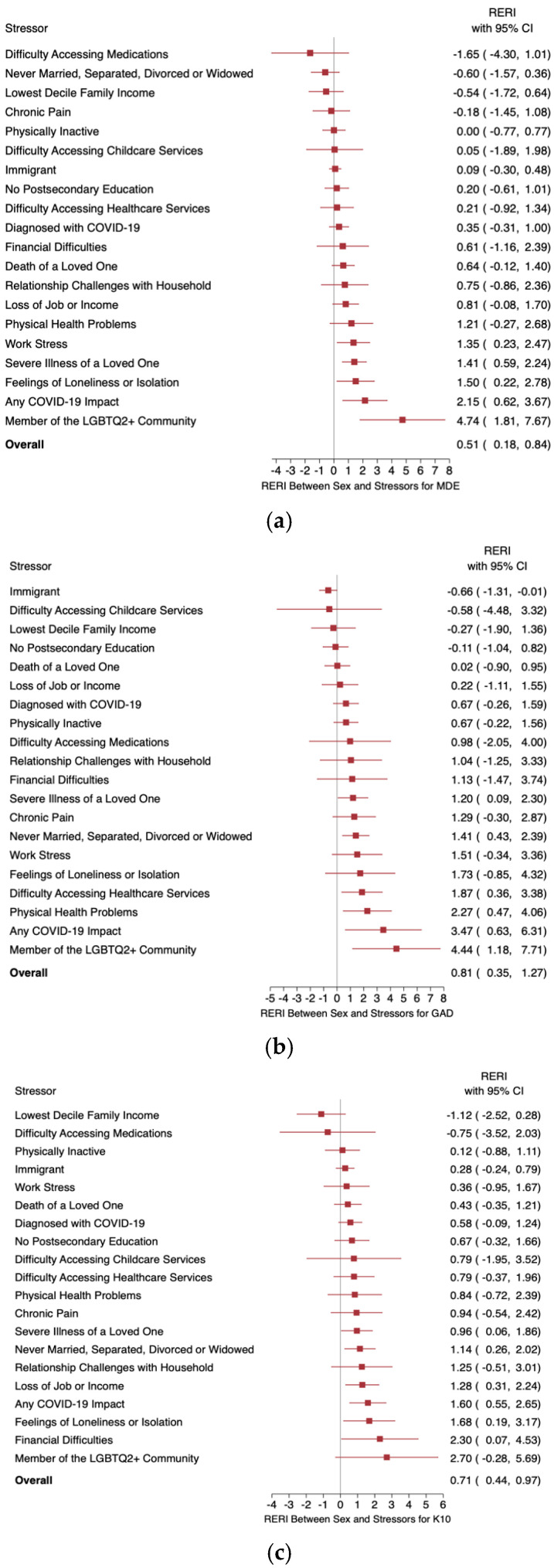
Forest plots summarizing the RERIs between sex and various pandemic-related stressors for (**a**) MDE, (**b**) GAD, and (**c**) K10 distress. Red squares represent the point estimates for the RERIs between sex and pandemic-related stressors; horizontal bars represent 95% CIs.

The RERIs between sex and each stressor for MDE, GAD, and distress are summarized in forest plots in Figure 2 and presented in more detail in Table 5. For MDE, there was a significant greater-than-additive interaction between sex and the following stressors: work stress, severe illness of a loved one, loneliness, and LGBTQ2+ status. For GAD, significant greater-than-additive interactions with age were shown for the severe illness of a loved one, marital status, difficulty accessing healthcare, and physical health problems. For K10 distress, significant greater-than-additive interactions with sex were found for the severe illness of a loved one, marital status, job/income loss, loneliness, and financial difficulties. Greater-than-additive interactions between the severe illness of a loved one, loneliness, marital status, and LGBTQ2+ status with sex were consistent for at least two outcomes. LGBTQ2+ status and the severe illness of a loved one showed a significant greater-than-additive interaction with age across all three outcomes. An experience of any COVID-19 impact showed greater-than-additivity with sex and with age, across all three outcomes.

## 4. Discussion

In this study, we aimed to explore the prevalence of MDE, GAD, and general psychological distress and evaluate the potential synergistic effect of pandemic-related stressors with age and sex within the general household population in Canada. Our findings indicate that young people and females experienced higher levels of MDE, GAD, and distress compared to older adults and males. The results provide, subject to the limitations of cross-sectional data, epidemiologic confirmation that youth and females have heightened vulnerability to pandemic-related stressors. Young females may be especially vulnerable to pandemic-related stressors and are predicted to have poorer mental health. Females who are part of the LGBTQ2+ community are also more likely to experience mental health challenges. Chief among our findings is that young females are especially vulnerable to developing MDE, GAD, or distress in future pandemics.

Consistent with suggestions contained in the extant literature [15], the prevalence of several COVID-19 impacts were greater in youth than in older adults and in females than in males. Age and sex differences based on higher rates of MDE, GAD, distress, and pandemic-related stressors alone may suggest greater vulnerability. However, this conclusion is subject to the limitations of cross-sectional data. While reverse causation is not a concern given the nature of these demographic variables, the results from MHACs reflect the prevalence, not risk or incidence, of the outcomes. The analysis assumes that the statistical interactions reflect causal synergy, but in the absence of temporal detail, other types of effects cannot be excluded, such as the possibility that pre-pandemic mental health problems may have caused some of the pandemic-related stressors, or that the synergies observed may reflect effects of the exposures on prognosis rather than risk. A critical limitation is that pre-existing mental health issues before the pandemic onset may have created pandemic-related stressors such as financial difficulties, rather than the reverse. Such a possibility cannot be ruled out without a longitudinal analysis. Many previous studies have speculated greater vulnerability by comparing prevalence differences before and during COVID-19 [55], but this study was able to examine interactions between pandemic-related stressors with age and sex. Such knowledge can encourage preventative efforts against MDE and GAD during pandemic periods, particularly with the value of targeting their delivery toward young people and females.

Our analysis draws on Rothman’s sufficient-component cause model. The relationship between statistical risk additivity and biological causation can be understood through this framework, which posits that most health outcomes arise from multiple combinations of component causes that collectively form causal mechanisms for disease [43,44,56,57]. Pandemic-related stressors are known to contribute to the development of mental health disorders [25,26,27,28,29,30,31,32,33,34,35,36,37,38,39], but their effects are not uniform across populations. Neither age nor sex alone is a sufficient determinant, as individuals within these groups may differ in their responses to such exposures. Instead, these stressors may interact with age or sex to form distinct causal pathways. If no pathways require the simultaneous presence of a specific stressor and a particular demographic factor (e.g., young age or female sex), the combined risk in the exposed group would reflect the sum of the individual risks. However, if certain causal mechanisms depend on the interaction of a stressor with age or sex, the joint risk will exceed the sum of the risks observed for each factor independently. Greater-than-additive interactions, therefore, suggest that some young people or females exposed to pandemic-related stressors are at heightened risk of developing mood or anxiety disorders specifically due to the synergies that arise because they are both young and have been exposed to pandemic-related stressors or are both female and have been exposed to pandemic-related stressors.

Generally, evidence of synergy was found between age and pandemic-related stressors, and between sex and pandemic-related stressors. Some additional RERIs, though their lower confidence bounds cut below zero, are consistent with the greater-than-additivity seen in the RERIs whose lower confidence bound exceeds zero, suggesting the hypothesis of a more general tendency in the direction of synergy than is confirmed statistically at the level of precision attained in the MHACS. The CIs for these variables include RERIs far above zero, and thus should not be interpreted as showing that these stressors do not show synergy. Furthermore, almost all pandemic-related stressors had RERI models with age and sex whose point estimates exceeded 1.0. For the interaction between age and pandemic-related stressors for MDE, these included the severe illness of a loved one, job/income loss, and chronic pain. For GAD, these included interactions between age with chronic pain and loneliness. For K10 distress, these included interactions between age with chronic pain, LGBTQ2+ status, physical health problems, loneliness, and difficulty accessing healthcare. For the interaction between sex and pandemic-related stressors for MDE, these included the death of a loved one, relationship challenges with household members, job/income loss, physical health problems, and LGBTQ2+ status. For GAD, these included chronic pain, work stress, and loneliness. For K10 distress, these included interactions between sex with difficulty accessing healthcare services, physical health problems, chronic pain, relationship challenges with household members, and LGBTQ2+ status. Thus, while not conclusive, the evidence suggests that age and sex may have an overall synergistic relationship with pandemic-related stressors.

Notably, the loss of social connection was associated with high levels of MDE, GAD, and distress in young people. Loneliness and familial relationship challenges had the strongest associations with poor mental health, and the RERIs between age and loneliness and between age and relationship challenges were consistently higher than for other pandemic-related stressors. The RERI between age and relationship challenges for psychological distress was the highest significant RERI seen among the pandemic-related stressors. This is consistent with the general sentiment that loneliness was a concomitant feature of social distancing, quarantining, and other pandemic restrictions [32,33]. School closures forced students to isolate themselves at home, preventing them from socializing with peers and connecting with friends, which may have led to their greater vulnerability to loneliness. Furthermore, problems with relationships in households have also cut off youth from loved ones. These circumstances may have contributed to their feelings of loneliness and isolation, which negatively impacted their mental health. Being isolated—physically or emotionally—from social groups has been shown to produce negative mood states and anxiety [17]. Our findings indicate that these outcomes may have been exacerbated in young people, especially as loneliness and relationship challenges emerged in the home. Disruption of important social interactions or facilitation of harmful relationship issues should be considered in decision-making associated with future public health mandates.

Females may also experience greater vulnerability to pandemic stressors. Interestingly, females were subject to excess risk for depression when exposed to pandemic stressors related to social losses, and for psychological distress when exposed to pandemic stressors related to both social losses and economic difficulties. The RERIs between sex and job/income loss and between sex and financial difficulties were among the highest for distress. The RERIs between sex and loneliness and between sex and the severe illness of a loved one were among the highest for depression. This supports the idea that social isolation and disruptions in social interactions may have had a greater impact on females [20]. Disruptions to social interactions and support networks disproportionately affect women, who benefit more from positive social relationships and are more vulnerable to a lack thereof [58]. That is, females, who are more reliant on social connectivity and support for emotional well-being, are especially vulnerable to social losses. Social losses reduced access to typical coping mechanisms, while the severe illness of a loved one intensified caregiving burdens. These stressors likely amplified the mental health toll on females as pandemic situations break down mechanisms of social support and relational dependencies, which are crucial for mitigating psychological stress and promoting positive mental health.

The greater vulnerabilities seen in youth and females require considerations for primary prevention. Given the strong associations between various pandemic-related stressors and MDE, GAD, and distress, and the increased vulnerability of youth to social isolation and females to social isolation and economic challenges, preparations for future pandemic responses that minimize the extent of these impacts specifically on these vulnerable groups become necessary. Social losses and economic hardships are both modifiable determinants that can be addressed using effective public policies and programs [59]. Examples of targeted interventions for youth that may mitigate the effects of pandemic-related stressors include increasing student participation in efforts to continue schooling during pandemic shifts to maintain social connection and peer socialization. Robust peer support programs should also be established as a layer of social support. We recommend further research on how to best support these population groups during pandemic crises. Overall, the development of comprehensive upstream interventions against the various social determinants of depression and anxiety is crucial to mitigating the differential mental health burden on young people and females. Bolstering social and economic supports will have especially beneficial impacts.

Our findings also indicate a need to highlight the differential effects of other stressors. The RERI between sex and LGBTQ2+ for MDE and GAD was large, insofar as it noticeably surpassed all other stressors. Thus, females who are part of the LGBTQ2+ community were significantly more likely to experience greater vulnerability to poor mental health. LGBTQ2+ females have compounding identities that create interdependent systems of disadvantage and may lead to greater inequities during pandemics. LGBTQ2+ females experience various unique mechanisms that lead to greater vulnerability to poorer mental health. The strong association found between LGBTQ2+ status and sex are consistent with the idea that internal psychosocial stress exacerbates experiences of external stressors, especially in individuals with intersecting minority identities [60]. Such internal stress that exacerbates mental health challenges includes identity concealment, expectations of rejection and prejudice, and the internalization of negative societal views like homophobia [60,61,62,63,64]. Additionally, the RERI between age and work stress indicated a greater vulnerability to poor mental health among youth facing work stress. This supports the idea that mental health challenges among youth were potentially exacerbated by prolonged and recurrent school and workplace closures during the pandemic period, with heightened anxieties and stress surrounding schedule changes, overwhelming academic demands, and uncertainty about the future [11].

Several limitations arising in this study must be highlighted. The cross-sectional design of the MHACS is one of these. A longitudinal analysis of the long-term impacts of COVID-19 remains necessary to examine the long-term association of pandemic-related stressors with youth and female mental health. For example, the long term effects of pandemic-related stressors after the removal of restrictions needs to be further explored, such that one study showed that poor mental health remained high among young people and females even after the end of the emergency [65]. Though the MHACS is comparable to mental health surveys conducted in Canada, such as the 2012 Canadian Community Health Survey, future research must contain baseline estimates of MDE, GAD, and distress to highlight temporal trends of these mental health outcomes. The lack of temporal data prevents the robust exploration of the causality of pandemic-related stressors. Also, while the MHACS included a validated diagnostic interview, such interviews are not considered as accurate as a detailed clinical assessment. Similarly, the pandemic-related stressors were evaluated using ad hoc items rather than validated instruments, which may have introduced reporting bias or inaccuracies due to recall issues, social desirability bias, or variability in how participants interpreted stressors. The response rate to the MHACS, when household and individual responses are considered, was only about 25%. However, this was at least partially addressed by adjustments for non-response made to the StatCan sampling weights. These adjustments ensured that weights were redistributed to maintain strong representation of the Canadian general household population. Additionally, Statistics Canada conducted a bias evaluation comparing MHACS estimates to 2021 Census benchmarks, which demonstrated minimal deviation, with nearly 85% of estimates within 1% and all estimates within 2% of their benchmarks. These provide strong evidence that non-response adjustments effectively reduced bias in the weighted data. Nevertheless, it must be acknowledged that underrepresented groups could have potentially experienced higher levels of pandemic-related stressors and may experience different patterns of synergy. Though representative of the general household population, the results should not be generalized to the groups excluded from the sampling frame. Further research examining the effects of the pandemic among Indigenous populations is recommended.

## 5. Conclusions

This study helps to identify population groups that are more vulnerable to the adverse effects of a public health emergency. As hypothesized, there is a general tendency for a synergistic effect of pandemic-related stressors with young age and female sex. Pandemic-related stressors may be associated with increased vulnerability to poor mental health in youth, females, and especially young females. Whether in Canada or similar developed nations, this information will be useful in the event of future pandemic events and other public health emergencies. Our findings should be compared with future longitudinal studies to strengthen assessments regarding causality and temporality. Jurisdictions must consider the negative consequences of the public health response seen during COVID-19, especially the greater vulnerability of young females to poor mental health when exposed to such consequences. Such considerations can encourage improvements to public health efforts in primary and secondary prevention.

## Figures and Tables

**Table 1:** Demographic characteristics of the population under study in MHACS in 2022.

Demographic	Frequency, % (95% CI)
Sex, female, %	51.06 (50.97–51.14)
Age group, %	
15–24 years	14.09 (14.09–14.09)
25+ years	85.91 (85.91–85.91)
Marital status, %	
Married or common law	56.97 (56.41–57.52)
Never married, widowed, separated, or divorced	43.03 (42.48–43.59)
Education level, %	
≤High school graduation	35.53 (34.98–36.07)
>Some post-secondary education	64.47 (63.93–65.02)
Immigrant status, %	30.09 (29.61–30.57)
LGBTQ2+ status, %	6.54 (6.23–6.85)

**Table 2:** Frequency estimates of various mental health outcomes, demographic characteristics, and COVID-19 pandemic-related and other stressors by population group.

Variable	Frequency, % (95% CI)				
	Total Population	15–24 Age Group	25+ Age Group	Females	Males
Mental health outcomes					
Past 12-month MDE	7.56 (7.22–7.90)	13.45 (12.52–14.38)	6.59 (6.22–6.96)	9.24 (8.72–9.77)	5.80 (5.37–6.24)
Past 12-month GAD	5.21 (4.93–5.50)	8.37 (7.66–9.08)	4.69 (4.38–4.99)	6.75 (6.31–7.20)	3.61 (3.25–3.96)
K10 psychological distress	6.26 (5.94–6.59)	10.62 (9.78–11.47)	5.55 (5.19–5.90)	7.52 (7.04–8.00)	4.96 (4.54–5.39)
Demographic characteristics					
15–24 years of age	14.09 (14.09–14.09)	100	0	13.41 (13.33–13.48)	14.82 (14.75–14.90)
Female	51.06 (50.97–51.14)	48.54 (48.26–48.83)	51.47 (51.39–51.56)	100	0
Lowest decile family income	10.28 (9.92–10.66)	6.52 (5.91–7.14)	10.91 (10.49–11.32)	11.23 (10.70–11.76)	9.32 (8.80–9.85)
Marital status	43.03 (42.48–43.59)	94.28 (93.62–94.94)	34.64 (33.99–35.38)	44.00 (43.20–44.81)	42.04 (41.22–42.86)
No post-secondary education	35.53 (34.98–36.07)	71.08 (70.00–72.17)	29.74 (29.13–30.35)	33.55 (32.82–34.28)	37.61 (36.83–38.38)
Immigrant status	30.09 (29.61–30.57)	22.14 (21.26–23.01)	31.37 (30.84–31.93)	29.61 (28.97–30.26)	30.62 (29.92–31.33)
LGBTQ2+ status	6.54 (6.23–6.85)	16.37 (15.33–17.41)	4.95 (4.64–5.26)	7.64 (7.18–8.11)	5.39 (4.97–5.81)
Pandemic-related stressors					
Loss of job or income	18.86 (18.38–19.35)	20.81 (19.71–21.92)	18.54 (18.01–19.08)	18.39 (17.71–19.07)	19.34 (18.63–20.05)
Financial difficulties	13.52 (13.07–13.96)	12.89 (11.94–13.83)	13.62 (13.12–14.11)	12.78 (12.16–13.41)	14.29 (13.66–14.91)
Difficulty accessing childcare	4.09 (3.83–4.35)	0.69 (0.45–0.94)	4.64 (4.34–4.94)	4.48 (4.10–4.86)	3.69 (3.34–4.03)
Difficulty accessing medications	4.56 (4.30–4.81)	3.09 (2.66–3.51)	4.80 (4.51–5.09)	5.09 (4.72–5.47)	4.01 (3.66–4.36)
Difficulty accessing healthcare	19.18 (18.69–19.66)	13.74 (12.83–14.64)	20.07 (19.53–20.62)	21.64 (20.93–22.35)	16.60 (15.94–17.27)
Diagnosed with COVID-19	22.13 (21.59–22.66)	30.52 (29.22–31.82)	20.75 (20.17–21.33)	22.08 (21.38–22.78)	22.17 (21.42–22.93)
Severe illness of a loved one	22.66 (22.14–23.18)	22.13 (21.04–23.22)	22.75 (22.16–22.33)	23.10 (22.36–23.84)	22.20 (21.44–22.97)
Death of a loved one	19.80 (19.30–20.29)	17.05 (16.09–18.02)	20.25 (19.70–20.80)	20.42 (19.72–21.12)	19.15 (18.46–19.84)
Feelings of loneliness or isolation	40.25 (39.65–40.85)	52.22 (50.94–53.51)	38.29 (37.62–38.96)	46.89 (46.02–47.76)	33.35 (32.52–34.18)
Physical health problems	22.49 (21.96–23.01)	23.66 (22.57–24.74)	22.29 (21.72–22.86)	26.53 (25.76–27.30)	18.29 (17.59–19.00)
Household relationship challenges	18.49 (18.01–18.98)	22.70 (21.62–23.78)	17.81 (17.26–18.35)	21.18 (20.46–21.91)	15.71 (15.07–16.36)
Any COVID-19 impact *	75.68 (75.16–76.20)	81.04 (80.02–82.06)	74.80 (74.22–75.37)	78.02 (77.32–78.72)	73.24 (72.48–74.00)
Other stressors					
Work stress	69.47 (68.68–70.16)	62.27 (60.76–63.78)	70.83 (70.06–71.60)	74.01 (73.06–74.96)	65.21 (64.23–66.19)
Chronic pain	24.37 (23.85–24.89)	13.69 (12.76–14.62)	26.12 (25.54–26.72)	27.17 (26.42–27.91)	21.48 (20.77–22.19)
Physical inactivity	21.64 (21.14–22.15)	13.87 (12.95–14.79)	22.91 (22.34–23.49)	23.13 (22.40–23.87)	20.06 (19.36–20.76)

* The ‘any’ COVID-19 impact variable was created by combining the pandemic-related stressor items included, as described above.

**Table 3:** Odds ratios for demographic characteristics, pandemic-related stressors, and other stressors as risk factors for past-12-month MDE, GAD, and general psychological distress.

Variable	Odds Ratio (95% CI) *		
	MDE	GAD	Distress
Demographic characteristics			
15–24 years of age	**2.20 (1.81–2.68)**	**1.86 (1.49–2.31)**	**2.02 (1.63–2.51)**
Female	**1.65 (1.35–2.02)**	**1.94 (1.52–2.47)**	**1.56 (1.25–1.94)**
Lowest decile family income	**1.44 (1.07–1.94)**	**1.55 (1.08–2.23)**	**1.44 (1.04–2.00)**
Marital status	1.10 (0.82–1.49)	0.99 (0.69–1.43)	1.19 (0.84–1.69)
No postsecondary education	**1.62 (1.33–1.97)**	1.22 (0.95–1.58)	**2.06 (1.65–2.58)**
Immigrant status	**0.56 (0.44–0.71)**	**0.50 (0.37–0.67)**	0.83 (0.65–1.06)
LGBTQ2+ status	**4.82 (3.68–6.32)**	**4.38 (3.20–5.99)**	**4.85 (3.61–6.53)**
Pandemic-related stressors			
Loss of job or income	**1.75 (1.40–2.19)**	**1.78 (1.36–2.33)**	**1.81 (1.42–2.32)**
Financial difficulties	**3.03 (2.41–3.80)**	**3.22 (2.44–4.24)**	**3.99 (3.11–5.12)**
Difficulty accessing childcare	**1.71 (1.11–2.63)**	**2.82 (1.78–3.46)**	**2.51 (1.60–3.93)**
Difficulty accessing medications	**2.71 (1.91–3.84)**	**2.86 (1.97–4.16)**	**3.10 (2.17–4.43)**
Difficulty accessing healthcare	**2.29 (1.85–1.84)**	**2.78 (2.16–3.58)**	**2.49 (1.96–3.16)**
Diagnosed with COVID-19	1.23 (0.99–1.53)	1.30 (1.00–1.70)	1.14 (0.88–1.47)
Severe illness of a loved one	**1.97 (1.61–2.42)**	**1.85 (1.45–2.35)**	**1.88 (1.47–2.40)**
Death of a loved one	**1.52 (1.21–1.89)**	1.16 (0.88–1.53)	**1.35 (1.04–1.75)**
Feelings of loneliness or isolation	**4.77 (3.84–5.92)**	**5.85 (4.39–7.79)**	**5.67 (4.27–7.54)**
Physical health problems	**4.09 (3.34–5.00)**	**3.96 (3.12–5.04)**	**3.95 (3.15–4.95)**
Household relationship challenges	**3.84 (3.11–4.74)**	**4.02 (3.13–5.15)**	**4.26 (3.35–5.43)**
Any COVID-19 impact	**4.68 (3.10–7.07)**	**5.36 (3.18–9.05)**	**3.69 (2.34–5.83)**
Other stressors			
Work stress	**2.68 (2.00–3.61)**	**2.69 (1.85–3.78)**	**2.56 (1.76–3.72)**
Chronic pain	**2.56 (2.11–3.11)**	**2.97 (2.34–3.78)**	**3.48 (2.78–4.36)**
Physical inactivity	**1.33 (1.06–1.67)**	1.29 (0.98–1.70)	**1.74 (1.38–2.20)**

* For all reported ORs, *p* < 0.001; bold font indicates significance given the 95% CIs.

**Table 4:** RERIs between young age and various stressors by mental health outcome.

Stressor	RERI with Age (95% CI)		
	MDE	GAD	K10 Distress
Demographic characteristics			
Lowest decile family income	−0.26 (−2.04, 1.53)	−0.43 (−2.30, 1.44)	−1.30 (−2.73, 0.13)
Marital status	25.01(−21.80, 71.82)	−0.95 (−3.17, 1.27)	−2.11 (−4.94, 0.72)
No postsecondary education	0.17 (−0.68, 1.01)	−1.00 (−2.01, 0.02)	0.08 (−0.89, 1.05)
Immigrant status	0.13 (−0.50, 0.77)	−0.26 (−0.88, 0.36)	−0.27 (−1.01, 0.47)
LGBTQ2+ status	0.24 (−2.44, 2.92)	2.84 (0.02, 5.65)	1.44 (−1.33, 4.21)
Pandemic-related stressors			
Loss of job or income	1.33 (−0.02, 2.68)	0.83 (−0.50, 2.17)	0.53 (−0.77, 1.83)
Financial difficulties	0.56 (−1.63, 2.75)	0.19 (−2.12, 2.50)	−1.76 (−4.20, 0.68)
Difficulty accessing medications	1.32 (−2.56, 5.19)	4.51 (−1.36, 10.38)	4.71 (−1.26, 10.68)
Difficulty accessing healthcare	2.30 (0.14, 4.45)	2.80 (0.32, 5.27)	2.22 (−0.02, 4.45)
Diagnosed with COVID-19	0.02 (−0.83, 0.87)	0.04 (−0.91, 0.99)	−0.47 (−1.31, 0.37)
Severe illness of a loved one	1.27 (−0.10, 2.63)	0.90 (−0.44, 2.24)	0.69 (−0.60, 1.99)
Death of a loved one	0.45 (−0.80, 1.69)	0.46 (−0.63, 1.56)	−0.05 (−1.23, 1.12)
Feelings of loneliness or isolation	2.15 (0.24, 4.07)	2.23 (−0.05, 4.51)	1.85 (−0.41, 4.11)
Physical health problems	2.41 (0.09, 4.73)	0.14 (−2.04, 2.32)	1.53 (−0.63, 3.69)
Household relationship challenges	3.12 (0.91, 5.32)	2.67 (0.36, 4.98)	4.00 (1.48, 6.52)
Any COVID-19 impact	4.14 (1.63, 6.65)	4.00 (1.40, 6.60)	2.62 (0.74, 4.50)
Other stressors			
Work stress	2.79 (0.64, 4.93)	0.65 (−2.19, 3.49)	2.74 (0.70, 4.78)
Chronic pain	2.29 (−0.34, 4.91)	1.27 (−1.23, 3.77)	1.32 (−1.75, 4.39)
Physical inactivity	0.68 (−0.74, 2.11)	0.27 (−1.07, 1.62)	0.23 (−1.37, 1.83)

**Table 5:** RERIs between female sex and various stressors by mental health outcome.

Stressor	RERI with Sex (95% CI)		
	MDE	GAD	K10 Distress
Demographic characteristics			
Lowest decile family income	−0.54 (−1.72, 0.64)	−0.27 (−1.90, 1.36)	−1.12 (−2.52, 0.28)
Marital status	−0.60 (−1.57, 0.36)	1.41 (0.43, 2.39)	1.14 (0.26, 2.02)
No postsecondary education	0.20 (−0.61, 1.01)	−0.11 (−1.04, 0.82)	0.67 (−0.32, 1.66)
Immigrant status	0.09 (−0.30, 0.48)	−0.66 (−1.31, −0.01)	0.28 (−0.24, 0.79)
LGBTQ2+ status	4.74 (1.81, 7.67)	4.44 (1.18, 7.71)	2.70 (−0.28, 5.69)
COVID-19 pandemic-related stressors			
Loss of job or income	0.81 (−0.08, 1.70)	0.22 (−1.11, 1.55)	1.28 (0.31, 2.24)
Financial difficulties	0.61 (−1.16, 2.39)	1.13 (−1.47, 3.74)	2.30 (0.07, 4.53)
Difficulty accessing childcare	0.05 (−1.89, 1.98)	−0.58 (−4.48, 3.32)	0.79 (−1.95, 3.52)
Difficulty accessing medications	−1.65 (−4.30, 1.01)	0.98 (−2.05, 4.00)	−0.75 (−3.52, 2.03)
Difficulty accessing healthcare	0.21 (−0.92, 1.34)	1.87 (0.36, 3.38)	0.79 (−0.37, 1.96)
Diagnosed with COVID-19	0.35 (−0.31, 1.00)	0.67 (−0.26, 1.59)	0.58 (−0.09, 1.24)
Severe illness of a loved one	1.41 (0.59, 2.24)	1.20 (0.09, 2.30)	0.96 (0.06, 1.86)
Death of a loved one	0.64 (−0.12, 1.40)	0.02 (−0.90, 0.95)	0.43 (−0.35, 1.21)
Feelings of loneliness or isolation	1.50 (0.22, 2.78)	1.73 (−0.85, 4.32)	1.68 (0.19, 3.17)
Physical health problems	1.21 (−0.27, 2.68)	2.27 (0.47, 4.06)	0.84 (−0.72, 2.39)
Household relationship challenges	0.75 (−0.86, 2.36)	1.04 (−1.25, 3.33)	1.25 (−0.51, 3.01)
Any COVID-19 impact	2.15 (0.62, 3.67)	3.47 (0.63, 6.31)	1.60 (0.55, 2.65)
Other stressors			
Work stress	1.35 (0.23, 2.47)	1.51 (−0.34, 3.36)	0.36 (−0.95, 1.67)
Chronic pain	−0.18 (−1.45, 1.08)	1.29 (−0.30, 2.87)	0.94 (−0.54, 2.42)
Physical inactivity	0.00 (−0.77, 0.77)	0.67 (−0.22, 1.56)	0.12 (−0.88, 1.11)

## Data Availability

The data used in this study are available from Statistics Canada in their Research Data Centers and accessible to those with approved research projects. Statistics Canada has also released a Public Use Microdata File (PUMF) for the Mental Health and Access to Care Survey (MHACS), which includes many safeguards to prevent the identification of any one person or household. The MHACS PUMF can be accessed at https://www150.statcan.gc.ca/n1/en/catalogue/82M0021X (Accessed 19 January 2025).
